# Awareness regarding the epidemiology, prevention, treatment, and consequences of viral hepatitis among a predominantly young adult population in the Podillia region (Ukraine): a cross-sectional study

**DOI:** 10.1016/j.pmedr.2026.103644

**Published:** 2026-09-20

**Authors:** Iryna Paliy, Dmytro Palii, Sergii Melashchenko, Olena Voinalovych, Olena Haiduk, Oleh Ksenchyn, Inna Chernova

**Affiliations:** a Department of internal and family medicine, National Pirogov Memorial Medical University, Vinnytsia, 21050, Mahistrats'ka street, b.44 – CPMSD#2, Ukraine; b Department of Epidemiology, National Pirogov Memorial Medical University, Vinnytsia, 23222, Berezyna village, Kashtanova street, b.5 – Clinical Center for Infectious Diseases, Ukraine

**Keywords:** Viral hepatitis, Public health, Public awareness, Health literacy, Epidemiology, Prevention, Podillia region, Ukraine

## Abstract

**Objective:**

To assess awareness of viral hepatitis A (HAV), B (HBV), and C (HCV) epidemiology, prevention, and treatment among adults in the Podillia Region (Ukraine), and identify knowledge predictors to address barriers to hepatitis elimination**.**

**Methods:**

An anonymous online cross-sectional survey evaluated 655 respondents (December 2024–May 2025) using a weighted multilevel scoring questionnaire. Separate Total Knowledge Scores (TKS) for HAV, HBV, and HCV were calculated. Predictors were assessed via multiple linear regression.

**Results:**

Inadequate knowledge (<60% score) was highly prevalent: 63.4% for HAV, 61.1% for HBV, and 68.8% for HCV. Comprehensive understanding of HCV transmission was significantly lower than for HAV and HBV (*p* < 0.01). Positive independent predictors of HCV knowledge included Internet use (B = 5.32), affected relatives (B = 4.53), physicians (B = 3.96), school (B = 3.09), parents (B = 2.73), and older age (B = 0.11) (all *p* < 0.05). Male sex independently predicted lower awareness across all three models (HCV: B = −3.60, *p* < 0.01).

**Conclusions:**

Viral hepatitis awareness is critically insufficient, particularly regarding HCV. Digital platforms and healthcare professionals are key knowledge determinants. Targeted educational interventions should prioritize these channels and specifically engage the male population to support elimination goals.

## Highlights

• Substantial viral hepatitis knowledge gaps in the Podillia region may reflect a broader health literacy challenge in Ukraine.
• Knowledge gaps were greatest for hepatitis C transmission and curability.
• Internet use and healthcare professionals were important predictors of higher knowledge.
• Men consistently demonstrated lower hepatitis knowledge scores than women.
• Targeted digital health education may help address hepatitis knowledge gaps.

## Graphical abstract

**Figure:**
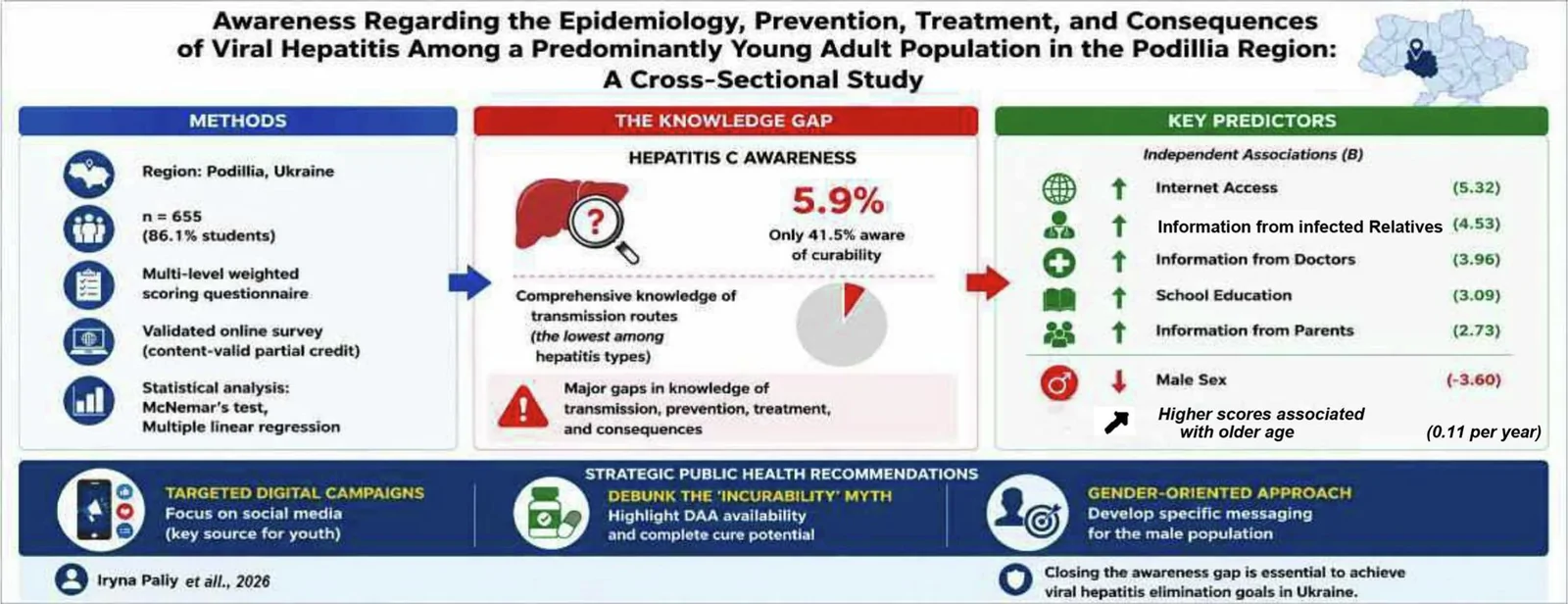

## 1 Introduction

Viral hepatitis A (HAV), B (HBV), and C (HCV) represent distinct public health challenges because of substantial differences in their transmission, natural history, prevention, treatment, and long-term consequences. HAV is primarily transmitted via the fecal-oral route, causing acute, self-limiting infections. In contrast, HBV and HCV are blood-borne infections that may become chronic, leading to cirrhosis and hepatocellular carcinoma. According to the 2024 WHO Global Hepatitis Report, approximately 254 million people live with chronic HBV and 50 million with HCV. In 2022, HBV and HCV accounted for 1.3 million deaths globally (1.1 million from HBV and 240,000 from HCV), predominantly due to cirrhosis and hepatocellular carcinoma (World Health Organization, 2024). Transmission risks also differ: HAV is acquired via contaminated food/water and close contact, whereas HBV and HCV are transmitted through infected blood, unsafe medical procedures, or sharing injection equipment; HBV is also transmitted sexually and perinatally. HAV, by contrast, contributes substantially less to global hepatitis mortality but remains important because of its potential to cause outbreaks and acute morbidity. Importantly, the three infections also differ substantially in their prevention and treatment options: HAV and HBV are vaccine-preventable, chronic HBV infection can be effectively controlled with long-term antiviral therapy, whereas HCV infection is curable with modern direct-acting antivirals (DAAs). Despite these advances, major gaps remain in the cascade of care: only 13% of people living with chronic HBV infection and 36% of those with HCV infection are aware of their status, limiting timely access to treatment and efforts to interrupt transmission (World Health Organization, 2024). These epidemiological, clinical, preventive, and therapeutic differences emphasize the need to consider knowledge of HAV, HBV, and HCV separately when evaluating public awareness and progress toward the 2030 hepatitis elimination goals.

Low health literacy is a key modifiable barrier to effective screening (European Association for the Study of the Liver, 2020). In Ukraine, viral hepatitis detection has also been affected by major disruptions to healthcare services. During the COVID-19 pandemic, reduced access to testing was accompanied by a marked decline in the reported incidence of chronic HBV and HCV, followed by an increase as testing activity recovered (Koval et al., 2025).The full-scale war has introduced additional barriers through disruption of routine healthcare services, redirection of healthcare resources, population displacement, and reduced access to diagnostic facilities. Recent epidemiological data illustrate the distinct patterns of the three infections. In 2024, the reported incidence of chronic HBV and HCV in Ukraine reached 4.0 and 18.5 cases per 100,000 population, respectively (Koval et al., 2025). HAV has a different epidemiological pattern: over the past three decades, its epidemic process in Ukraine has been characterized predominantly by sporadic cases and occasional localized outbreaks, including outbreaks involving up to approximately 350 cases (Palii et al., 2025). Recent nationally representative seroepidemiological data further suggest that the burden of HBV and HCV infection in Ukraine may exceed that reflected by routinely reported incidence statistics (Kasatkina et al., 2025). The same national study assessed hepatitis D virus (HDV) markers specifically in the context of HBV infection; HDV is therefore not considered here as an independent epidemiological entity but as an infection that occurs in association with HBV (Kasatkina et al., 2025).

Insufficient public awareness affects the prevention and management of all three viral hepatitis infections, although the relevant knowledge gaps differ by virus. For HCV, misconceptions regarding curability remain an important psychological barrier to testing despite the availability of highly effective DAA therapy (Torre et al., 2024; Ward et al., 2020). For HBV, inadequate understanding of transmission routes, the risk of chronic infection, and the importance of vaccination may undermine prevention and timely diagnosis. For HAV, awareness of fecal–oral transmission, hygiene, sanitation, and vaccination is particularly important for outbreak prevention. Thus, public awareness should not be considered as a single construct for “viral hepatitis” but should reflect the distinct transmission patterns, preventive strategies, and treatment options of HAV, HBV, and HCV. Accordingly, this study aimed to assess knowledge among youth and adults in the Podillia region (Ukraine) regarding the transmission, epidemiology, clinical course, modern treatment modalities, and prevention of viral hepatitis A, B, and C.

## 2 Methods

### 2.1 Study design and setting

This anonymous cross-sectional online survey was conducted in the Podillia region of Ukraine (Vinnytsia and Khmelnytskyi oblasts) from December 2024 to May 2025 using Microsoft Forms. The study assessed knowledge of HAV, HBV, and HCV epidemiology, transmission, prevention, consequences, and treatment. Reporting followed the STROBE guidelines for cross-sectional studies (von Elm et al., 2007).

### 2.2 Study population, eligibility criteria, and sampling

Eligible participants were individuals aged ≥17 years residing in the Podillia region who provided informed consent; those with medical education were excluded. Participants were recruited by non-probability snowball sampling. The questionnaire link was distributed through social media and personal networks, with assistance from members of the Vinnytsia Regional Association of Family Physicians and academic staff of non-medical higher education institutions. Participants were encouraged to further distribute the link.

This pragmatic approach facilitated recruitment under martial law constraints and inclusion of internally displaced persons residing in the region (Kim et al., 2026; Naderifar et al., 2017). No formal a priori sample-size calculation was performed; the study aimed to recruit the largest feasible sample during the predefined study period. Overall, 660 questionnaires were submitted.

### 2.3 Questionnaire development, structure, and validation

The questionnaire comprised 30 questions covering sociodemographic characteristics, information sources, and knowledge of HAV, HBV, and HCV. Seven experts assessed its face and content validity, followed by pilot testing in 15 participants. Internal consistency was acceptable (Cronbach's α = 0.72, 0.73, and 0.73 for HAV, HBV, and HCV, respectively). The questionnaire is provided in Supplementary Appendix A.

### 2.4 Knowledge scoring and outcome measures

A weighted multilevel scoring system based on partial-credit principles was used (Betts et al., 2022; Zarei et al., 2024). Separate Total Knowledge Scores (TKS) were calculated for HAV (maximum 41), HBV (42), and HCV (43) and categorized using an adapted Bloom's cut-off (George et al., 2023) as high (≥80%), moderate (60–79%), or low (<60%). Comprehensive transmission knowledge required identification of all relevant routes without selecting incorrect options. Detailed item-level scoring and weighting criteria are provided in Supplementary Appendix B (Table B1).

### 2.5 Data collection and quality control

Participation was voluntary and anonymous, with no directly identifying information collected. Responses were not restricted to one per device because devices could be shared by eligible participants; therefore, duplicate responses could not be identified with certainty. Five of 660 questionnaires showing implausible response patterns (straightlining or repetitive pattern answering) were excluded, leaving 655 for analysis.

### 2.6 Ethical considerations

Participation was voluntary and anonymous, and all respondents provided informed consent. Under applicable local regulations, formal ethics committee approval was not required for this anonymous, non-interventional survey without identifiable health information (*look at* ***“Compliance with ethical standards” block*)**.

### 2.7 Statistical analysis

Continuous variables were summarized as means and standard deviations and categorical variables as frequencies and percentages. Paired proportions for HAV, HBV, and HCV were compared using McNemar's test. Separate multiple linear regression models identified predictors of HAV-, HBV-, and HCV-TKS, with age as a continuous variable and sex and information sources as categorical covariates; coefficients (B) with 95% confidence intervals were estimated.

Model diagnostics included residual histograms (Fig. 2), variance inflation factors (VIF), and the Breusch–Pagan test. All VIF values were < 3. HC3 robust standard errors were used for the heteroscedastic HAV model. Analyses were performed using Microsoft Excel 2013 and Python 3.6.15 (Pandas and Statsmodels), with two-sided *p* < 0.05 considered significant.

## 3 Results

### 3.1 Participant characteristics

After data-quality screening, five questionnaires with implausible response patterns were excluded, leaving 655 respondents for analysis. Mean age was 22.8 ± 10.2 years (range, 17–74), and 84.3% were aged 17–24 years. Females constituted 61.5% of the sample. Most respondents were non-employed pupils or university students (85.3%), while 13.3% were employed (Table 1). Given the non-probability snowball sampling and online recruitment, the sample was not demographically representative of the general population of the Podillia region.

**Table 1:** Socio-demographic characteristics of the validated cohort (n = 655) in the Podillia region (Ukraine), December 2024–May 2025.

Characteristic	Category	n	%
Age, years	Mean ± SD (range)	22.8 ± 10.2 (17–74)	–
	17–24	552	84.3
	25–34	29	4.4
	35–44	29	4.4
	45–54	24	3.7
	≥55	21	3.2

Sex	Female	403	61.5
	Male	252	38.5

Education	School pupil	11	1.7
	Vocational student	2	0.3
	University student	425	64.9
	Secondary education	68	10.4
	Secondary specialized/vocational	29	4.4
	Higher education	120	18.3

Occupational status	Student/pupil, not employed	559	85.3
	Employed⁎	87	13.3
	Unemployed/household/retired	9	1.4

Total		655	100.0

⁎ Participants who reported both studying and being employed were classified as employed.

### 3.2 Domain-specific knowledge of HAV, HBV, and HCV

Overall, 81.2% of respondents identified the liver as the primary organ affected by viral hepatitis, and 53.3% recognized HBV and HCV as blood-borne infections. In contrast, comprehensive knowledge of virus-specific transmission routes was substantially lower: 30.9% for HAV, 17.6% for HBV, and 5.9% for HCV (all pairwise *p* < 0.01) (Table 2).

**Table 2:** Domain-specific awareness among respondents in the Podillia region (Ukraine), December 2024–May 2025 (% of correct answers).

Parameter	HAV	HBV	HCV	*p*-value (A vs B)	p-value (A vs C)	p-value (B vs C)
Comprehensive transmission knowledge	30.9	17.6	5.9	<0.01	<0.01	<0.01
Knowledge of infection consequences	8.2	16.8	20.5	<0.01	<0.01	<0.01
Awareness of vaccination	53.5	53.5	32.6	0.93⁎	<0.01	<0.01
Awareness of curability	50.3	49.2	41.5	0.71⁎	<0.01	<0.01
Awareness of treatment availability	27.0	49.7	49.4	<0.01	<0.01	0.93⁎

⁎ Non-significant difference by McNemar's test with continuity correction. All other (significant) p-values were also obtained using McNemar's test.

Knowledge of infection consequences was also limited (HAV, 8.2%; HBV, 16.8%; HCV, 20.5%; all pairwise *p* < 0.01). Awareness of vaccination was 53.5% for both HAV and HBV and 32.6% for HCV, while correct knowledge of curability was 50.3%, 49.2%, and 41.5%, respectively. Awareness of treatment availability was lower for HAV (27.0%) than for HBV (49.7%) and HCV (49.4%) (both *p* < 0.01), with no difference between HBV and HCV (*p* = 0.93).

### 3.3 Virus-specific total knowledge scores

Mean TKS values were 21.8 ± 8.6 for HAV, 22.8 ± 8.7 for HBV, and 21.8 ± 9.2 for HCV. Low knowledge (<60% of the maximum score) predominated for all three infections: 63.4% for HAV, 61.1% for HBV, and 68.8% for HCV. High knowledge (≥80%) was observed in 12.8%, 13.6%, and 9.3%, respectively. (Table 3).

**Table 3:** Integral awareness indices among respondents in the Podillia region (Ukraine), December 2024–May 2025.

Index	HAV (max 41)	HBV (max 42)	HCV (max 43)
Mean score (M ± SD)	20.69 ± 9.60	21.75 ± 10.12	20.33 ± 9.99
High level, n (%)	84 (12.8%)	89 (13.6%)	61 (9.3%)
Moderate level, n (%)	156 (23.8%)	166 (25.3%)	143 (21.9%)
Low level, n (%)	415 (63.4%)	400 (61.1%)	451 (68.8%)

Moderate knowledge (60–79% of the maximum score) was observed in 23.8% of respondents for HAV, 25.3% for HBV, and 21.9% for HCV. High knowledge (≥80%) was relatively uncommon, accounting for 12.8%, 13.6%, and 9.3% of respondents for HAV, HBV, and HCV, respectively. Overall, HCV showed the largest proportion of respondents with low knowledge and the smallest proportion with high knowledge.

### 3.4 Factors associated with hepatitis knowledge

Multiple linear regression analysis identified several independent factors associated with virus-specific knowledge scores (Fig. 1*.*). Across all three models, Internet use, physicians, affected relatives, parents, and school as sources of information were independently associated with higher knowledge scores. Older age was also positively associated with knowledge, whereas male sex was consistently associated with lower scores. Traditional media, including television and newspapers, were not significant independent predictors.

**Fig. 1:**
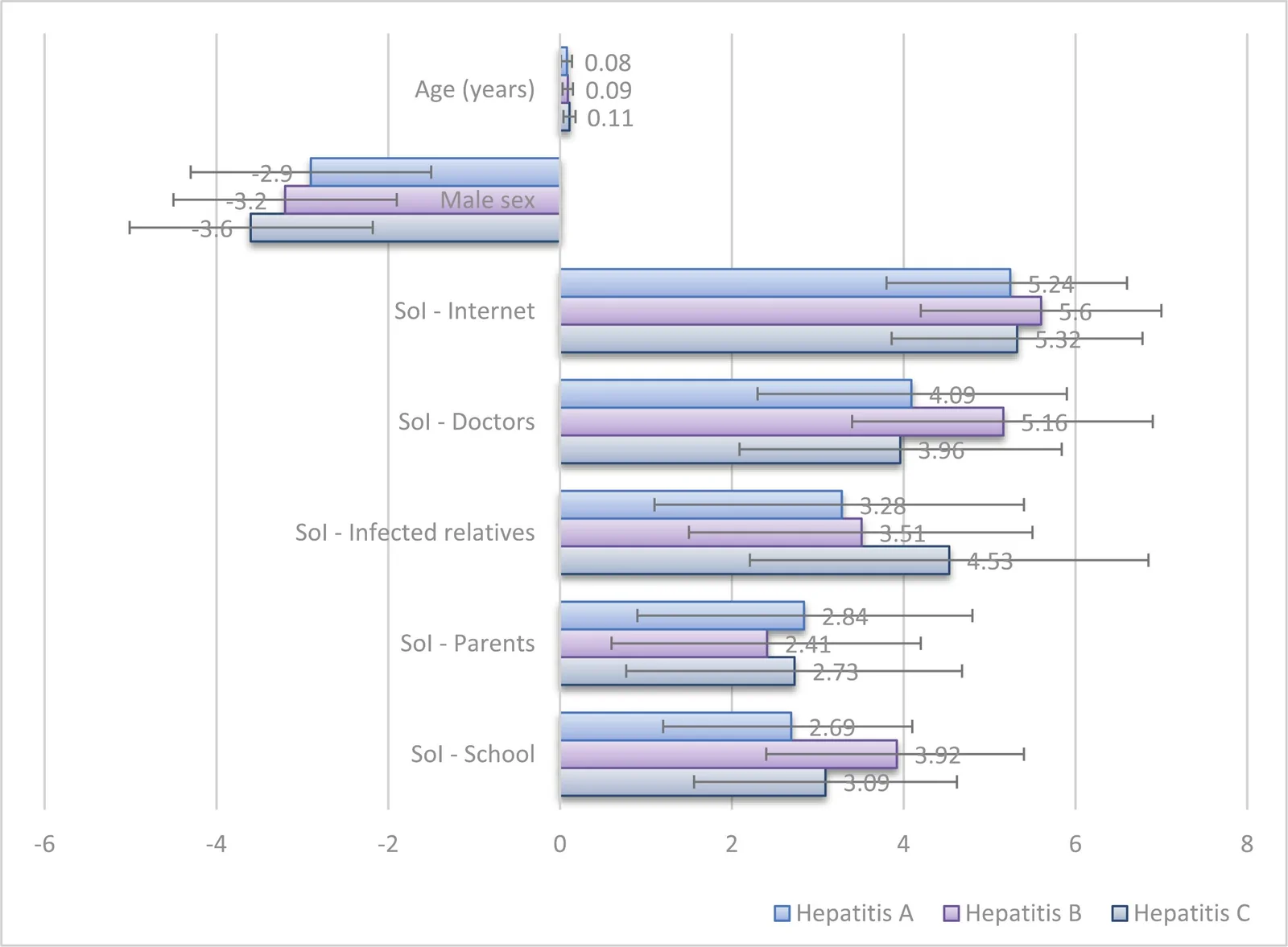
Independent predictors of knowledge regarding viral hepatitis A, B, and C identified by multiple linear regression analysis among respondents in the Podillia region (Ukraine), December 2024–May 2025. Points represent unstandardized regression coefficients (B), and horizontal error bars indicate 95% confidence intervals (95% CI). Positive coefficients indicate factors associated with higher knowledge scores, whereas negative coefficients indicate factors associated with lower knowledge scores. (SoI – Source of Information).

For HAV, Internet use showed the largest positive association with knowledge (B = 5.24, 95% CI: 3.8, 6.6), followed by physicians (B = 4.09, 95% CI: 2.3, 5.9), affected relatives (B = 3.28, 95% CI: 1.1, 5.4), parents (B = 2.84, 95% CI: 0.9, 4.8), and school (B = 2.69, 95% CI: 1.2, 4.1). Older age was positively associated with HAV knowledge (B = 0.08, 95% CI: 0.01, 0.14), whereas male sex was negatively associated with the score (B = −2.90, 95% CI: −4.3, −1.5).

Similar patterns were observed for HBV and HCV. For HBV, the strongest positive associations were observed for Internet use (B = 5.60, 95% CI: 4.2, 7.0) and physicians (B = 5.16, 95% CI: 3.4, 6.9), while male sex was associated with a 3.20-point lower score (95% CI: −4.5, −1.9). For HCV, Internet use (B = 5.32, 95% CI: 3.86, 6.78), affected relatives (B = 4.53, 95% CI: 2.21, 6.85), and physicians (B = 3.96, 95% CI: 2.09, 5.84) showed the largest positive associations. Male sex was associated with a 3.60-point lower HCV knowledge score (95% CI: −5.01, −2.18), while older age remained positively associated with knowledge (B = 0.11, 95% CI: 0.04, 0.18).

The models explained 23.1%, 26.5%, and 23.6% of the variance in HAV-, HBV-, and HCV-specific knowledge scores, respectively. Multicollinearity was low (all VIF values <3). Residual distributions were assessed visually using histograms (Fig. 2). The Breusch–Pagan test indicated heteroscedasticity in the HAV model (*p* = 0.04); therefore, HC3 robust standard errors were used for this model. No evidence of heteroscedasticity was observed for the HBV (*p* = 0.10) or HCV (*p* = 0.31) models.

**Fig. 2:**
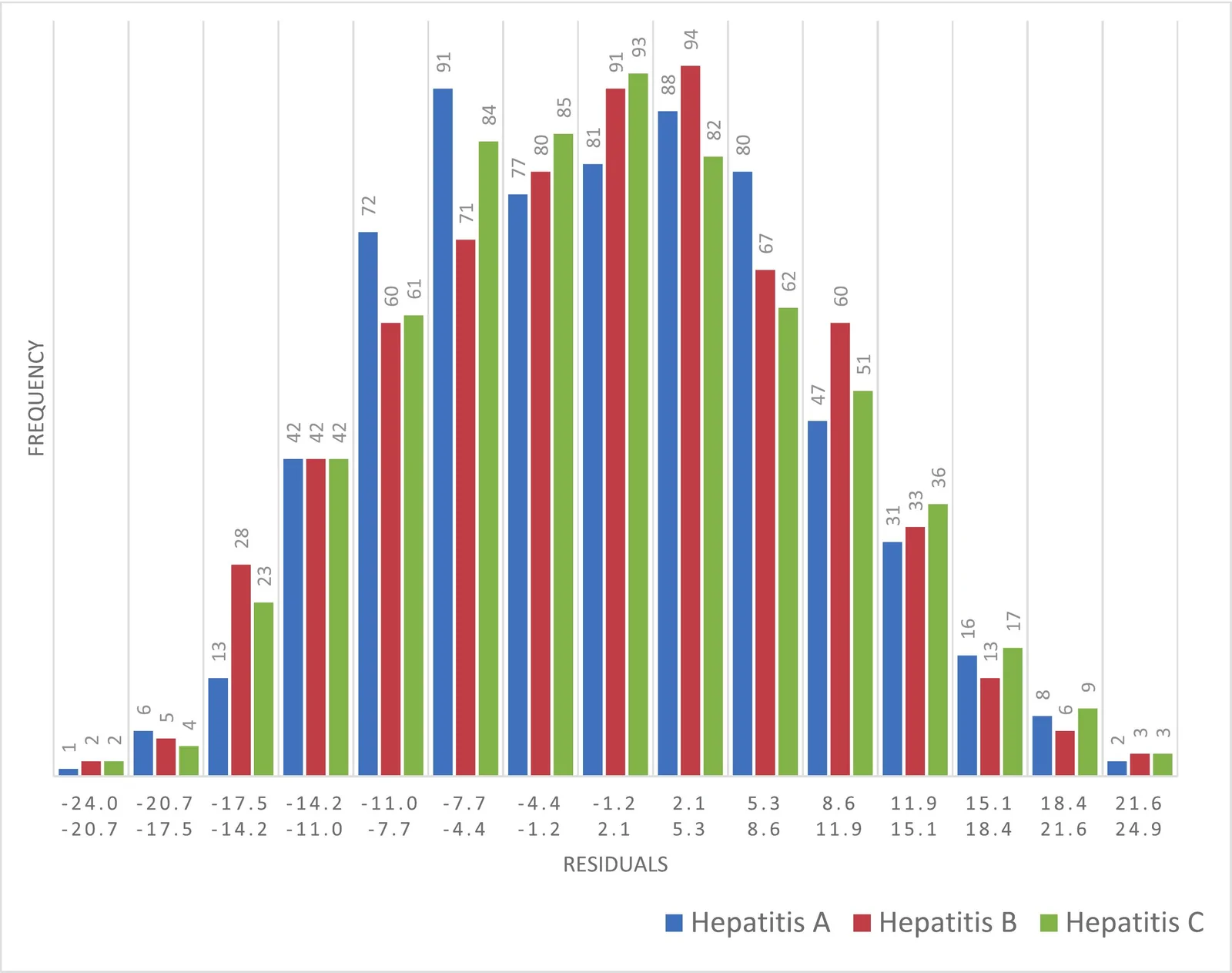
Histograms of residual distribution for multiple linear regression models evaluating knowledge regarding hepatitis A, B, and C among respondents in the Podillia region (Ukraine), December 2024–May 2025. The X-axis represents the difference between observed and predicted values (model residuals).

## 4 Discussion

To our knowledge, this is the first regional “Knowledge, Attitude, and Practice” (KAP) study in Ukraine to evaluate public awareness of HAV, HBV, and HCV separately using virus-specific scores. Our findings revealed substantial gaps across all three infections, with the greatest deficits observed for HCV. A broadly similar pattern was reported in a nationwide population-based survey in Greece, where high overall awareness was observed in 30.4% of respondents for HBV and 21.6% for HCV, with HCV showing the least favorable results (Kaskafetou et al., 2022). However, the Greek study defined a high level as 12–17 correct responses out of 17 items (≥70.6%), whereas our threshold was ≥80% of the corresponding virus-specific TKS, limiting direct numerical comparison.

Insufficient awareness of viral hepatitis has also been documented in neighboring Poland, which shares a broadly similar healthcare landscape with Ukraine (Ganczak et al., 2016), although the specific areas of misunderstanding differed across studies. Reports from Ethiopia and Saudi Arabia have likewise identified important gaps in awareness of viral hepatitis, but with considerable variation in the domains affected (Angelo et al., 2024) (Albadrani et al., 2023). Such cross-country comparisons should therefore be interpreted cautiously, as differences in reported levels may reflect not only genuine variation in population awareness but also differences in study populations, sampling strategies, questionnaire design and wording, response options, scoring systems, and thresholds used to categorize the results.

An important finding was the marked discrepancy between general recognition of blood-borne hepatitis and comprehensive understanding of HCV transmission. Although 53.3% of respondents correctly recognized HBV and HCV as primarily blood-borne infections, only 5.9% demonstrated comprehensive knowledge of HCV transmission routes according to the stricter virus-specific criterion. This suggests that familiarity with the general concept of blood-borne transmission does not necessarily translate into an accurate understanding of the individual routes through which HCV can be acquired.

The findings regarding HCV curability (41.5%) suggest that misconceptions remain an important barrier to screening. This is consistent with global data (Ward et al., 2020), but particularly relevant for Ukraine, where access to DAA treatment is supported through public health mechanisms. According to the analytical report of the Public Health Center (PHC), low awareness regarding the free availability of direct-acting antivirals is the primary barrier deterring patients from testing (Brandl et al., 2024). Achieving the WHO goal of diagnosing 90% of cases by 2030 will be difficult without targeted interventions (World Health Organization, 2022; World Health Organization, 2024).

Male sex was independently associated with lower knowledge scores for HAV, HBV, and HCV. Similar sex-related differences have been reported in some populations (Yakudima et al., 2022), whereas studies from other settings found no significant association between sex and hepatitis knowledge (Almalki et al., 2025; Mutaru et al., 2023).In our sample, men were less likely than women to report the Internet (54.0% vs 66.3%, *p* = 0.01) and teachers/school (26.2% vs 39.7%, *p* < 0.01) as sources of hepatitis information, whereas they more frequently reported acquaintances (31.0% vs 20.8%, *p* = 0.01). These differences in information sources may have contributed to the observed sex-related differences in knowledge; however, they do not fully explain them, as male sex remained independently associated with lower scores in multivariable models that included information sources. The reasons for this association therefore remain uncertain and may involve other unmeasured factors.

Internet use was independently associated with higher knowledge scores for all three hepatitis types, supporting the potential importance of digital information channels for hepatitis education. However, this association should not be attributed exclusively to the predominantly young composition of our sample, as Internet use is not limited to younger age groups. The high proportion of students and pupils in the study reflects the non-probability snowball recruitment strategy and should instead be considered when assessing the generalizability of our findings. Information obtained from physicians and affected relatives was also independently associated with higher knowledge scores across all three models, supporting the important role of healthcare professionals and personal experience as sources of hepatitis-related information.

Overall HCV knowledge remained limited: 68.8% of respondents had low knowledge (<60% of the maximum HCV-TKS), 21.9% had moderate knowledge (60–79%), and 9.3% reached the high-knowledge category (≥80%). These findings indicate substantial gaps in HCV-related awareness even within this predominantly young and highly educated sample and highlight the need for more effective public education on HCV transmission, prevention, and treatment.

The higher awareness of HAV may be partly explained by heightened public attention following the 2023 local outbreak in Vinnytsia (Palii et al., 2025).

### 4.1 Limitations

The cross-sectional design and snowball sampling limit causal inference and generalizability. The marked overrepresentation of young, female students constitutes a clear selection bias, indicating that our sample does not perfectly reflect the broader target population of the Podillia Region (Ukraine). Self-reporting may introduce information bias and social desirability bias (Hassan, 2006). The models explained only 23–27% of the variance, indicating the influence of additional unmeasured factors such as socio-economic status and personal beliefs. Despite these limitations, the study's strengths are the large sample size, the use of a multi-component validated approach, and multi-factor statistical modeling.

## 5 Conclusions

Population awareness regarding viral hepatitis A, B, and C in the Podillia region (Ukraine) is heterogeneous and insufficient, especially regarding HCV transmission and curability. Older age, Internet use, and consultation with doctors are key predictors of better knowledge. Male sex was a significant predictor of lower awareness, while traditional media demonstrated no impact. These findings suggest the need for targeted digital health interventions, particularly for men, to correct misconceptions and improve screening uptake.

## Authors statement

All authors made substantial contributions to the conception and design of the study, acquisition, analysis, or interpretation of data; participated in drafting the manuscript or revising it critically for important intellectual content; and approved the final version to be submitted. All authors agree to be accountable for their contributions to the work.

## Declaration of generative AI and AI-assisted technologies

In the manuscript preparation process. During the preparation of this manuscript, generative AI–assisted tools were used solely to support checking and improving English language, grammar, and clarity. The scientific content, interpretation of data, and conclusions were entirely the responsibility of the authors, who reviewed and approved the final manuscript.

## CRediT authorship contribution statement

**Iryna Paliy:** Writing – review & editing, Methodology, Conceptualization. **Dmytro Palii:** Supervision, Methodology, Conceptualization. **Sergii Melashchenko:** Writing – review & editing, Writing – original draft, Supervision, Formal analysis, Data curation. **Olena Voinalovych:** Project administration, Funding acquisition, Data curation. **Olena Haiduk:** Project administration, Data curation. **Oleh Ksenchyn:** Project administration, Formal analysis, Data curation. **Inna Chernova:** Project administration, Data curation.

## Compliance with ethical standards

The study was conducted in accordance with the Declaration of Helsinki and local institutional requirements. Participation was anonymous and voluntary. All respondents provided electronic informed consent. In accordance with local regulations, formal ethics committee approval was not required for this anonymous, non-invasive survey.

## Ethics declaration

### Informed consent and patient details

Written informed consent to take part in the study and to publish the article has been obtained from all participants or their legal representatives. The privacy rights of participants have been observed.

### Studies in human

This study was performed in compliance with relevant laws, regulatory frameworks and guidelines where the research took place. This study was conducted in accordance with Declaration of Helsinki and local institutional requirements. Ethics committee approval was not required under relevant laws and institutional guidelines. Under the applicable institutional requirements of National Pirogov Memorial Medical University, Vinnytsya, formal ethics committee review was not required for this anonymous, non-interventional survey. Participation was voluntary, all respondents provided informed consent, and no directly identifiable personal or health information was collected.

## Declaration of competing interest

The authors declare that they have no known competing financial interests or personal relationships that could have appeared to influence the work reported in this paper.

## Data Availability

Data will be made available on request.

## Appendix A Supplementary data

Supplementary material 1 English-language version of the questionnaire used in the survey

Supplementary material 2 Response scoring criteria and weighting system used to calculate the virus-specific Total Knowledge Scores (TKS) for hepatitis A, B, and C.
