# Supplementary material for: Awareness regarding the epidemiology, prevention, treatment, and consequences of viral hepatitis among a predominantly young adult population in the Podillia region (Ukraine): a cross-sectional study
Source: Prev Med Rep. 2026 Sep 20;71:103644. doi: 10.1016/j.pmedr.2026.103644 (PMC13634348; doi:10.1016/j.pmedr.2026.103644)
Supplement: Supplementary file 1 [file mmc1.docx]

# **Supplementary Appendix A**

## Questionnaire Used in the Cross-Sectional Study on Viral Hepatitis Awareness in the Podillia Region of Ukraine

**Study title:** *Awareness Regarding the Epidemiology, Prevention, Treatment, and Consequences of Viral Hepatitis among a Predominantly Young Adult Population in the Podillia Region: A Cross-Sectional Study*

**First author:** Iryna Paliy

|  | Link to the MS Forms questionnaire used in the study (*in Ukrainian*):  <https://forms.office.com/Pages/ResponsePage.aspx?id=MNlGcyLKNkOOwX0RVBOT5E2qkgWNTGZOvwVEaCDo8VNUQUwzWFJJMDlHWFBLSzJRN0YwQVk0TEdOSS4u> |
| --- | --- |

# English Translation of the Original Questionnaire

## Section 1. Sociodemographic Characteristics

Start of Block: Background Information

Q1 How old are You (yeares)?

Q2 Your gender:

- Male
- Female
- Unspecified

Q3 Your place of residence:

- City
- Urban settlement (small town)
- Village
- Places of deprivation of liberty

Q4 Your education:

- Secondary
- Specialized secondary
- Higher
- Technical college student
- University student

Q5 Your Occupation:

- Schoolchild
- Student
- Worker
- Officer
- Businessman
- Military
- Unemployed
- Pensioner
- Prisoner
- Other (*type*)

# Section 2. General Awareness and Epidemiology

### Q6. Viral hepatitis is a disease of the:

- Liver
- Stomach
- Joints
- Lungs
- Heart
- Brain
- Does not exist
- I do not know

### Q7. Which types of hepatitis are primarily transmitted through blood?

- Hepatitis A (Botkin’s disease)
- Hepatitis B
- Hepatitis C
- Alcoholic hepatitis
- Drug-induced hepatitis
- Fatty liver disease
- Other
- I do not know

### Q8. How is hepatitis A transmitted?

- Through contaminated food
- Through contaminated water
- Poor personal hygiene (dirty hands)
- Close contact with an infected person
- Mother-to-child transmission during childbirth
- Unprotected sexual intercourse
- Blood transfusion
- Dental procedures
- Hairdressing salon services involving reusable instruments
- Tattooing / body piercing / blood exposure procedures
- Use of unsterile injection equipment during drug use
- I do not know
- Other

### Q9. How is hepatitis B transmitted?

- Through contaminated food
- Through contaminated water
- Poor personal hygiene (dirty hands)
- Close contact with an infected person
- Mother-to-child transmission during childbirth
- Unprotected sexual intercourse
- Blood transfusion
- Dental procedures
- Hairdressing salon services involving reusable instruments
- Tattooing / body piercing / blood exposure procedures
- Use of unsterile injection equipment during drug use
- I do not know
- Other

### Q10. How is hepatitis C transmitted?

- Through contaminated food
- Through contaminated water
- Poor personal hygiene (dirty hands)
- Close contact with an infected person
- Mother-to-child transmission during childbirth
- Unprotected sexual intercourse
- Blood transfusion
- Dental procedures
- Hairdressing salon services involving reusable instruments
- Tattooing / body piercing / blood exposure procedures
- Use of unsterile injection equipment during drug use
- I do not know
- Other

# Section 3. Clinical, Therapeutic, and Preventive Aspects

### Q 11. What are the possible consequences after hepatitis A infection?

- Acute hepatitis
- Chronic hepatitis
- Liver cirrhosis
- Liver cancer
- Complete recovery
- No risk of liver cirrhosis
- No risk of liver cancer
- I do not know

### Q 12. What are the possible consequences after hepatitis B infection?

- Acute hepatitis
- Chronic hepatitis
- Liver cirrhosis
- Liver cancer
- Complete recovery
- No risk of liver cirrhosis
- No risk of liver cancer
- I do not know

### Q13. What are the possible consequences after hepatitis C infection?

- Acute hepatitis
- Chronic hepatitis
- Liver cirrhosis
- Liver cancer
- Complete recovery
- No risk of liver cirrhosis
- No risk of liver cancer
- I do not know

### Q14. Can hepatitis A be completely cured?

- Yes
- No
- I do not know

### Q 15. Can hepatitis B be completely cured?

- Yes
- No
- I do not know

### Q 16. Can hepatitis C be completely cured?

- Yes
- No
- I do not know

### Q 17. Are there specific antiviral drugs for the treatment of hepatitis A?

- Yes
- No
- I do not know

### Q18. Are there specific antiviral drugs for the treatment of hepatitis B?

- Yes
- No
- I do not know

### Q 19. Are there specific antiviral drugs for the treatment of hepatitis C?

- Yes
- No
- I do not know

### Q 20. How long does antiviral treatment for hepatitis B usually last?

- Antiviral treatment is not performed
- 1 week
- 3 months
- 6 months
- 1 year
- More than 1 year
- Lifelong
- I do not know

### Q 21. How long does antiviral treatment for hepatitis C usually last?

- Antiviral treatment is not performed
- 1 week
- 1 month
- 3–6 months
- 1 year
- More than 1 year
- Lifelong
- I do not know

### Q 22. What do you estimate the cost of antiviral treatment for hepatitis C to be?

- 100 UAH
- 1,000 UAH
- 50,000 UAH
- 100,000 UAH
- 500,000 UAH
- I do not know

### Q 23. Is there a vaccine against hepatitis A?

- Yes
- No
- I do not know

### Q 24. Is there a vaccine against hepatitis B?

- Yes
- No
- I do not know

### Q 25. Is there a vaccine against hepatitis C?

- Yes
- No
- I do not know

### Q 26. From what sources did you get information about viral hepatitis?

- Newspapers, magazines
- Television
- Internet forums
- Friends told me
- Parents conducted the conversation
- Teachers/lecturers conducted the conversation at the educational institution
- Someone close to you was sick
- Information from a doctor
- This is the first time I hear about such a disease

# Section 4. Social Attitudes and Behavioral Aspects

### Q 27. Would you avoid close contact with a person who has recovered from viral hepatitis?

- Yes
- No
- I do not know

### Q 28. Would you avoid eating at a place where kitchen staff had previously recovered from hepatitis?

- Yes
- No
- I do not know

### Q 29. Can individuals who recovered from hepatitis become blood donors?

- Yes
- No
- I do not know

### Q 30. Would you avoid being treated by a family physician who had previously recovered from viral hepatitis?

- Yes
- No
- I do not know

### Q 31. Would you avoid dental treatment from a dentist who had previously recovered from viral hepatitis?

- Yes
- No
- I do not know

### Q 32. Can individuals who recovered from hepatitis visit public swimming pools?

- Yes
- No
- I do not know

# Notes

- The original questionnaire was administered electronically using Microsoft Forms.
- The questionnaire was anonymous and voluntary.
- The original language of the questionnaire was Ukrainian.
- This document represents the English translation used for manuscript preparation and supplementary materials.
- Scoring criteria and weighting coefficients are described below
