# Supplementary material for: Awareness regarding the epidemiology, prevention, treatment, and consequences of viral hepatitis among a predominantly young adult population in the Podillia region (Ukraine): a cross-sectional study
Source: Prev Med Rep. 2026 Sep 20;71:103644. doi: 10.1016/j.pmedr.2026.103644 (PMC13634348; doi:10.1016/j.pmedr.2026.103644)
Supplement: Supplementary file 2 [file mmc2.docx]

# **Supplementary Appendix B**

Response Scoring Criteria

**Study title:** *Awareness Regarding the Epidemiology, Prevention, Treatment, and Consequences of Viral Hepatitis among a Predominantly Young Adult Population in the Podillia Region: A Cross-Sectional Study*

**First author:** Iryna Paliy

Table B1. **Questionnaire items and response scoring criteria**

| Question | Response Options | Scoring Criteria |
| --- | --- | --- |
| 6. Viral hepatitis is a disease of the: | Liver, Stomach, Joints, Lungs, Heart, Brain, Does not exist, I don't know | «5» – Liver; If «Liver» and any other option – minus 1 point; If without «Liver» – «1» point |
| 7. Which types of hepatitis are primarily transmitted through the blood? | A (Botkin's disease); B; C; Alcoholic; Drug-induced; Fatty; Other; I don't know | «5» – B and C; «4» – only B or C selected; «3» – B, C + one other option; «2» – B or C + one other option; «1» – any combinations where B or C are not selected |
| 8. How is hepatitis A transmitted? | Through contaminated food (1); Through contaminated water (2); Poor personal hygiene (e.g., dirty hands) (3); Close contact with an infected person (4); Mother-to-child transmission during childbirth (5); Unprotected sexual intercourse (6); Blood transfusion (7); Dental procedures (8); Hairdressing salon services with reusable instruments (shaving, manicure, pedicure, etc.) (9); Unsafe medical and other procedures involving blood exposure (tattooing, body piercing, etc.) (10); Use of unsterile injection equipment during drug use (11); I don't know (12); Other (13) | «+1.25» points for each correct item (1, 2, 3, 4); «–0.22» points for each incorrect item (5, 6, 7, 8, 9, 10, 11, 12, 13). The total score is rounded to the nearest whole number. Maximum «5» points, a negative result is recorded as «0» |
| 9. How is hepatitis B transmitted? | *(Same options as in Question 3)* | «+0.7» points for each correct item (6, 7, 8, 9, 10, 11); «–1» point for each incorrect item (1, 2, 3, 4, 13) or «–0.5» points for (5, 12). The total score is rounded to the nearest whole number. Maximum «5» points, a negative result is recorded as «0» |
| 10. How is hepatitis C transmitted? | *(Same options as in Question 3)* | «+0.7» points for each correct item (4, 6, 7, 8, 9, 10, 11); «–1» point for each incorrect item (1, 2, 3, 13) or «–0.5» points for (5, 12). The total score is rounded to the nearest whole number. Maximum «5» points, a negative result is recorded as «0» |
| 11. What are the potential consequences for a person after exposure to the hepatitis A virus? | Acute hepatitis (1); Chronic hepatitis (2); Risk of developing liver cirrhosis (3); Risk of developing liver cancer (4); Complete recovery (5); No risk of developing liver cirrhosis (6); No risk of developing liver cancer (7); I don't know (8) | «+1.25» points for each correct item (1, 5, 6, 7); «–0.25» points for each incorrect item (2, 3, 4, 8). The total score is rounded to the nearest whole number. Maximum «5» points, a negative result is recorded as «0» |
| 12. What are the potential consequences for a person after exposure to the hepatitis B virus? | Acute hepatitis (1); Chronic hepatitis (2); Risk of developing liver cirrhosis (3); Risk of developing liver cancer (4); Complete recovery (5); No risk of developing liver cirrhosis (6); No risk of developing liver cancer (7); I don't know (8) | «+1» point for each correct item (1, 2, 3, 4, 5); «–0.30» points for each incorrect item (6, 7, 8). The total score is rounded to the nearest whole number. Maximum «5» points, a negative result is recorded as «0» |
| 13. What are the potential consequences for a person after exposure to the hepatitis C virus? | Acute hepatitis (1); Chronic hepatitis (2); Risk of developing liver cirrhosis (3); Risk of developing liver cancer (4); Complete recovery (5); No risk of developing liver cirrhosis (6); No risk of developing liver cancer (7); I don't know (8) | «+1» point for each correct item (1, 2, 3, 4, 5); «–0.30» points for each incorrect item (6, 7, 8). The total score is rounded to the nearest whole number. Maximum «5» points, a negative result is recorded as «0» |
| 14. Can hepatitis A be completely cured? | Yes; No; I don't know | «5» – yes; «0» – I don't know; «0» – no |
| 15. Can hepatitis B be completely cured? | Yes; No; I don't know | «5» – no; «0» – I don't know; «0» – yes |
| 16. Can hepatitis C be completely cured? | Yes; No; I don't know | «5» – yes; «0» – I don't know; «0» – no |
| 17. Are there specific antiviral drugs for the treatment of hepatitis A? | Yes; No; I don't know | «5» – no; «0» – I don't know; «0» – yes |
| 18. Are there specific antiviral drugs for the treatment of hepatitis B? | Yes; No; I don't know | «5» – yes; «0» – I don't know; «0» – no |
| 19. Are there specific antiviral drugs for the treatment of hepatitis C? | Yes; No; I don't know | «5» – yes; «0» – I don't know; «0» – no |
| 20. How long do you think antiviral treatment for hepatitis B lasts? | Antiviral treatment is not performed (1); 1 week (2); 3 months (3); 6 months (4); 1 year (5); More than 1 year (6); Lifelong (7); I don't know (8) | «1» – options 6 or 7; «0» – all other options |
| 21. How long do you think antiviral treatment for hepatitis C lasts? | Antiviral treatment is not performed (1); 1 week (2); 1 month (3); 3-6 months (4); 1 year (5); More than 1 year (6); Lifelong (7); I don't know (8) | «1» – option 4 (3-6 months); «0» – all other options |
| 22. What do you estimate the cost of such treatment to be? | 100 UAH; 1,000 UAH; 50,000 UAH; 100,000 UAH; 500,000 UAH; I don't know | «1» – 50,000 UAH; «0» – all other options |
| 23. Is there specific prophylaxis (a vaccine) against hepatitis A? | Yes; No; I don't know | «5» – yes; «0» – I don't know; «0» – no |
| 24. Is there specific prophylaxis (a vaccine) against hepatitis B? | Yes; No; I don't know | «5» – yes; «0» – I don't know; «0» – no |
| 25. Is there specific prophylaxis (a vaccine) against hepatitis C? | Yes; No; I don't know | «5» – no; «0» – I don't know; «0» – yes |
| 27. Would you avoid close contact with a person who has recovered from viral hepatitis? | Yes; No; I don't know | «1» – no; «0» – I don't know; «0» – yes |
| 28. Would you avoid eating at establishments where it is known that the kitchen staff have recovered from hepatitis? | Yes; No; I don't know | «1» – no; «0» – I don't know; «0» – yes |
| 29. Can individuals who have recovered from hepatitis be blood donors? | Yes; No; I don't know | «1» – no; «0» – I don't know; «0» – yes |
| 30. Would you avoid follow-up and treatment by a family physician who has recovered from viral hepatitis? | Yes; No; I don't know | «1» – no; «0» – I don't know; «0» – yes |
| 31. Would you avoid treatment by a dentist who has recovered from viral hepatitis? | Yes; No; I don't know | «1» – no; «0» – I don't know; «0» – yes |
| 32. Can individuals who have recovered from hepatitis visit swimming pools? | Yes; No; I don't know | «1» – yes; «0» – I don't know; «0» – no |
